# Glycosyltransferase Gene Expression Profiles Classify Cancer Types and Propose Prognostic Subtypes

**DOI:** 10.1038/srep26451

**Published:** 2016-05-20

**Authors:** Jahanshah Ashkani, Kevin J. Naidoo

**Affiliations:** 1 Scientific Computing Research Unit, Faculty of Science, University of Cape Town, Rondebosch, 7701, South Africa; 2 Department of Chemistry, Faculty of Science, University of Cape Town, Rondebosch, 7701, South Africa.

## Abstract

Aberrant glycosylation in tumours stem from altered glycosyltransferase (GT) gene expression but can the expression profiles of these signature genes be used to classify cancer types and lead to cancer subtype discovery? The differential structural changes to cellular glycan structures are predominantly regulated by the expression patterns of GT genes and are a hallmark of neoplastic cell metamorphoses. We found that the expression of 210 GT genes taken from 1893 cancer patient samples in The Cancer Genome Atlas (TCGA) microarray data are able to classify six cancers; breast, ovarian, glioblastoma, kidney, colon and lung. The GT gene expression profiles are used to develop cancer classifiers and propose subtypes. The subclassification of breast cancer solid tumour samples illustrates the discovery of subgroups from GT genes that match well against basal-like and HER2-enriched subtypes and correlates to clinical, mutation and survival data. This cancer type glycosyltransferase gene signature finding provides foundational evidence for the centrality of glycosylation in cancer.

Glycosylation is the major posttranslational modification (PTM) in cellular development. Added to this is the central role played by glycoconjugates in cell-cell communication. Structural alterations to complex carbohydrate (glycans) structures represent a key signature in the development of neoplastic character in the proliferation cells. Structural alterations of glycans on normal cells that are on a neoplastic transformation path to malignancy have been documented over the last few decades^1^. The transformation requires neoplastic cells to first invade surrounding cells before they can metastasize. Here cancer related oligosaccharide changes have been implicated with the invasive properties of cancer cells^2^. Further glycans have been shown to play a central role in metastasis of for example breast cancer^3^. While these altered glycans make promising candidates for cancer biomarker discovery and indeed most early FDA approved markers are either glycans or glycoconjugates^4^, progress is limited as their *in vivo* detection poses serious challenges.

The term cancer has come to describe complex malignant diseases that may not share the same causative agents, etiology or molecular profiles^5^. Cancer related oligosaccharide changes have been associated with hallmarks underpinning tumour cell death prevention or cell proliferation^2^,^4^. Alterations in oligosaccharide structures are due to the expressions of enzymes that make up the glycosylation machinery, particularly glycosyltransferases (GTs). The expression levels of these enzymes are controlled by dysregulation at the transcriptional level, dysregulation of chaperone function as well as altered glycosidase activity^4^. The differential changes to cellular glycan structures are predominantly regulated by the expression patterns of glycosyltransferase genes and are a hallmark of neoplastic cell metamorphoses. It is accepted that individual enzymes responsible for alterations in glycan structures could be biomarkers^6^, however a comparison of the collective enzymatic actions between cancers leading to type or subtype specific glycosylation profile definitions have not been considered.

We found evidence that the changes of glycan structures are strongly implicated as signatures in malignant tumour typing and possibly subtyping. This PTM orchestrated by the regulation of GT genes and the subsequent biochemical action of glycosyltransferases engineers the restructuring of glycans that in turn play key roles in the progression toward malignancy reliant on tumorigenesis^7^,^8^,^9^,^10^,^11^. Here we probed the relationship between expression levels of 210 GT genes and cancer type by examining the expression data of 1893 samples, representing six cancer types (breast invasive carcinoma; BRCA, ovarian serous cystadenocarcinoma; OV, glioblastoma multiforme; GBM, kidney renal clear cell carcinoma; KIRC, colon adenocarcinoma; COAD and lung squamous cell carcinoma; LUSC).

## Results and Discussion

**Figure 1:**
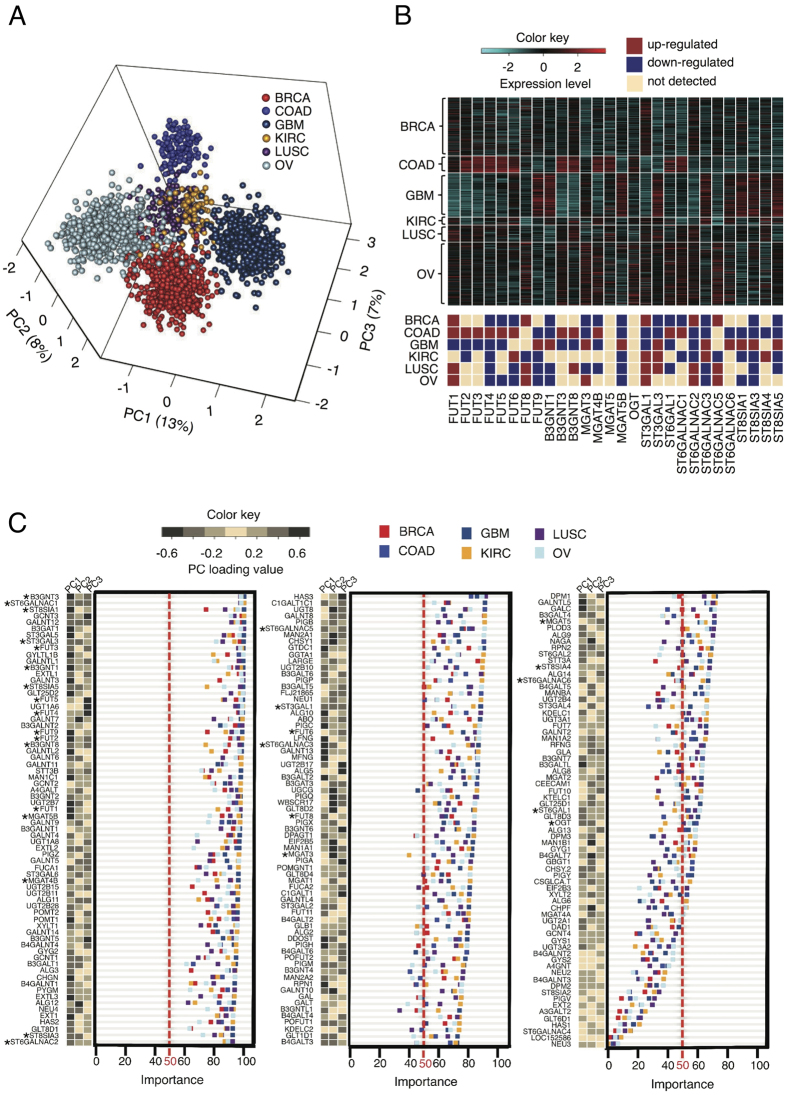
The expression profile of 210 GT genes segregates six cancer types. To separate cancer types based on the expression of GT genes, a principal component analysis was performed and to better understand how the expression of glycosyltransferase genes contribute to the separation of cancer types from each other and to investigate dominant glycan-specific changes that occur in the carcinogeneic process of each cancer type. The expressions of glycosyltransferase genes were compared amongst the cancer types and the association of GT genes to patient survival was studied. (**A**) A principal component analysis of six tumour types using expressions of GT genes demonstrates a capability to separate six cancer types including breast: breast invasive carcinoma [BRCA, n = 531], ovary: ovarian serous cystadenocarcinoma [OV, n = 578], brain: glioblastoma multiforme [GBM, n = 403], kidney: kidney renal clear cell carcinoma [KIRC, n = 72], colon: colon adenocarcinoma [COAD, n = 154] and lung: lung squamous cell carcinoma [LUSC, n = 155]). (**B**) Expression profile of the GT genes known to be important to tumourigenesis in six cancer types. Genes with q-value ≤ 0.005 and 2 fold change were considered as a differentially expressed gene in pairwise comparisons. Top panel shows the GT gene expressions for each cancer type for a selection of known genes linked to key phenotypes. These GT genes display differential expression (bottom panel) in different cancer types when compared to each other. (**C**) Plot of gene importance in identification of six cancer types for GT genes important for tumourigenesis using a model-based approach (i.e. pam). The red broken line is a border for genes with importance of 50 or more for identifying cancers. Significance level of PC loading values (PC1-PC3) is shown adjacent to the importance plot.

A principal component analysis (PCA)^12^ of the expression of GT genes in six tumor types was performed to measure the ability of the GT genes to segregate cancer types. The first three principal components (PC1–PC3) account for a 28% variation between the six tumor types and separated them into six clearly demarcated groups (Fig. 1A). A hierarchical average linkage clustering performed across all the samples revealed that the expression profiles of GT genes between the cancer types are significantly altered and that breast cancer basal-like is unique molecular entity (Fig. S2). While it is established that the biological pathways of all cancer types are a shared feature^13^ it is not clear whether the glyco-biochemical system of events correlated to each pathway in every cancer is the same. Consequently, the importance of GT genes in the supervised classification of cancer types was examined in relation to their ability to identify cancer types (Fig. 1C).

GT genes have known linkages to the key phenotypes apoptosis, motility, epidermal growth factor receptor (EGFR) tyrosine kinase, angiogenesis, invasion and adhesion that define tumour malignancy. The underlying rationale for differential GT gene expression levels in six cancers is better understood through an understanding of the roles that the translated enzymes play in the reprogramming of the integrated intercellular circuitry and the sub circuits supporting tumour cell-biological properties. We discuss the regulation patterns in relation to pathways that affect cell fate and metastasis.

### Viability, Cytostasis and Differentiation Circuits

The β3Gnt family of enzymes plays significant roles in colon, brain and ovarian cancer progression. Here the highest ranked important GT gene for the identification of cancer type is β3GNT3. Correspondingly β3GNT3 has high PC1 loading values for the segregation of cancers (Fig. 1C). The overexpression of this gene has recently been shown to suppress T antigen formation and was proposed as a diagnostic marker of neuroblastoma^14^, and the enzyme that it encodes is a marker for the early detection of ovarian cancer^15^. T antigens inhibit the p53 and Rb family of tumor suppressors subsequently over expression of β3GNT3 should be investigated for links to averting cell death in tumors. Family members β3GNT2 and β3GNT8 appear in the top 50 most important genes (Fig. 1C) and are known for their roles in cancer in contrast to β3GNT1 that has not yet been fingered as significant.

The expression levels of the four main families of sialyltransferases (ST3Gal (α2,3 linkage), ST6Gal (α2,6 linkage), ST6GalNAc (α2,6 linkage), and ST8Sia (α2,8 linkage)) can vary between different tumor cases and tumor types. Increased sialylation critically affects the viability and cytostasis circuits in determining tumour cell fate. Different cells express different antigens and may exhibit coexpression of these antigens on individual cells such that Tn, STn, T, and normal core 1 based structures can all be expressed on the same tumor. In comparison, benign lesions rarely coexpress multiple antigens^16^. In the case of T antigens (T, sT, STn) in BRCA, LUSC and OV the ST3GAL1 and ST6GALNAC2 genes are relatively up-regulated. The ST3Gal-I enzyme has been shown to mask the galactose residues with sialic acids in O-glycans and glycolipids^17^. Generally O-linked glycans are frequently truncated in tumours, often as a result of premature sialylation where the sialyltransferase ST3Gal-I transfers N-acetylneuraminic acid (SA) via α2–3 linkage to the galactose residue in core 1 to form sialyl core-1 or sialyl T. ST3Gal-I sialyltransferase is required for core 1 O-glycan sialylation on CD8+ T cells and its deficiency induces core 2 O-glycan biosynthesis. In the case of core 1 O-glycan sialylation deficiency apoptosis follows. ST6GALNAC2 (ranked within top third most important genes) is the more important of the two for supervised cancer classification. Furthermore, the variation in its expression levels between the six cancer types supports its importance ranking and its significance in the separation of the six cancer types.

### Motility Circuits and Metastasis

Aberrant sialylation during the biosynthesis glycosphingolipids (gangliosides) in the Golgi apparatus is important to cell adhesion and motility in many cancers. Gangliosides such as GD1c, GT1a, GQ1b, and GT3^18^ require their coded glycosyltransferases. One such gene, ST6GALNAC1 was observed here to be up-regulated in COAD and there are many LUSC and OV samples that overexpress this gene (Fig. 1B). It is the second most important ranked gene in the supervised identification of cancer types but not a significant role player in separating cancers (Fig. 1C). Genes such as ST8SIA1 and ST8SIA5 that are key to gangliosides synthesis are overexpressed in many samples of GBM and form part of the top 15 most important genes that lead to a GT gene classifier of the six cancer types.

The elevated presence of β1–6 branching on cell surface N-glycans observed in the molecular analysis of tumors is correlated with the up-regulation of N-glycan branching enzymes (MGATs) this is positively correlated with the histological grade and tumour node metastasis^19^,^20^,^21^,^22^. An increase in branching structures are not only associated with the initial stages of cancer but also with the progression to advanced stages and metastasis^23^,^24^.

MGAT5B encodes β1,6-N-acetylglucosaminyltransferase-5b, a glycosyltransferase (GnT-Vb or GnT-IX) that is an isozyme of GnT-V. While both enzymes synthesize the β(1,6)-GlcNAc linkage to α (1,6)-linked mannose on N-linked glycans the GnT-IX protein transfers GlcNAc to both the α1,3- mannose arm of the N-glycan as well. In addition GnT-IX transfers a GlcNAc in a β1,6-linkage to the mannose in GlcNAcβ1,2-Manα-O-Ser. Therefore GnT-IX enzyme acts on both N and O-glycans with the latter considered to be its primary target and considered to be a primary aberrant glycosylation in brain cancers^25^. Despite its perceived minor role across all cancers, MGAT5B has a high loading value in PC1 and is one of the top 50 most important genes affecting cancer identification (Fig. 1C).

Equally MGAT4B is highly ranked as an important gene affecting cancer classification and shows a decrease in most of investigated cancer types compared to OV and KIRC This demonstrates the critical role of MGAT4B in N-glycan branching on the surface of these tumour cells, however MGAT4B is widely expressed in most tissues^26^.

Intercellular receptors Integrins, Cadherins and Selectins underpin adhesion and are integral to the motility circuits and metastasis. Over expression of E-selectin-mediated adhesion of cancer cells to vascular endothelium, is central in the hematogenous metastasis of cancer cells. Sialyl lewis A (sLea) and sialyl lewis X (sLex) are selectin ligands that aid tumour cell adhesion to endothelia, platelets and leukocytes. sLea/x over-expression is an important event leading to metastasis. A critical component of sLea antigen synthesis is ST3Gal-III. Important to the different roles played by sLea/x in the metastatic pathways amongst cancers is the observation that the ST3GAL3 gene is very clearly over-expressed in GBM, is relatively up-regulated in KIRC and LUSC but relatively down-regulated in BRCA and COAD (Fig. 1B). This variable expression is encapsulated in the significant PC1 loading value that underlies the role it plays in the separation as well as being one of the top ten most important genes for supervised cancer classification (Fig. 1C).

Several fucosyltransferases (FUT) are involved in fucosylating cell surface glycan residues in a α2–3 and/or 4 linkages at the terminus of the N- and O-linked glycan structures. This leads to the expression of cancer-associated blood group Lewis antigens Lex/Ley and Lea/Leb. Reviewing the expression profiles of FUT gene family members revealed that they are highly varied amongst the cancer types and are over-expressed in many samples except GBM where they are under-expressed except for FUT9 (Fig. 1B). This rationalizes the significant PC1 and PC3 loading values (Fig. 1C) and underscores the important role they play in the separation of the six cancer types. Moreover, FUT1-5 as well as FUT9 forms part of the 50 most important GT genes for supervised cancer classification (Fig. 1C).

The major mechanism regulating SLea/x expression is the upregulation of sialyltransferases and not fucosylation. FUT3 is the major fucosyltransferase responsible for synthesizing SLea. However, with the exception of colon and kidney cancers, it is not usually up-regulated in tumors^27^ (Table S3). From the expression patterns reported here this gene is inversely expressed in COAD and GBM cancers, plays an important role in segregating cancer types and ranks in the top ten most important GT genes affecting the supervised cancer classification.

### Evaluation of GT Gene Classifier

**Figure 2:**
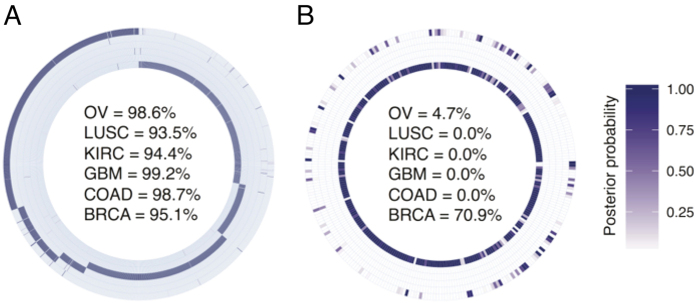
Evaluation of the classifier built using the expression of glycosyltransferase genes, which is able to identify cancer type from a random sample. For the purpose of error estimation of the training model (pam classifier) in the assignment of samples to the correct cancer type 10-fold cross validated, internal tests and an external tests were carried out. (**A**) Circular heatmap representing the results of internal tests. The glycosyltranseferases’ expression dataset was randomly split one hundred times into training (70%) and test (30%) sets. Training sets were used to build models that were then applied to the testing sets. The median values computed for each cancer type were used to assign each sample to a specific cancer type. The result of this analysis was used for accuracy measurement calculation summarized in Table S4. (**B**) Circular heatmap representing the results of the external test (GSE20624) containing 177 GT genes that are common to the TCGA data and comprising 293 breast cancer samples^29^. Percentage values (inside to outside) of samples correctly assigned to tumour type (in the centre of heat maps) with posterior probability ≥0.95. Sidebar represents the median value of posterior probability assigning each sample to a specific cancer type.

Using the shrunken centroid approach^28^ the development of a GT gene classifier which is able to identify cancer type from a random sample was explored. A10-fold cross validation of the classifier shows that all 210 GT genes are necessary to maintain a level of accuracy (>0.95) where the misclassification error is on average less than 0.02 (Table 1). The classifier is able to determine the overall identity of more than 95% of the test sample tumor type (Fig. 2B). A data repository (GSE20624) comprising 293 breast cancer samples^29^ provided independent verification of the classifier accuracy although only 177 GT genes are shared with the TCGA data. Here 70.9% samples are classified as breast cancer and 4.7% as ovarian cancer (Fig. 2C). GT gene expression presents an opportunity for cancer assessments as a means of identifying cancer types.

### Classification of Breast Cancer Subtypes

In addressing the ability of glycosyltransferase genes in breast cancer subtyping, we performed consensus average linkage clustering that allows a quantitative and visual assessment of the estimated number of unsupervised subtypes in a particular cancer^30^. This method employs the usual measure of judging the accuracy of a clustering experiment is the consideration of the extent to which there is intra-cluster variation (cluster compactness), the degree to which there is inter-cluster variation (cluster separation). Resampling techniques allow the evaluation of the stability and validity of the clusters. Specifically here we performed consensus average linkage clustering on BRCA data to discover subtypes. The results indicate that clustering stability increases from k = 2 to k = 10 (Fig. S3) and upon visual inspection of the clusters (Fig. S3 panel A) we observed that five clusters (k = 5) produced the most compact and clearly separated clustering. This may explain the heterogeneity observed in breast tumors, while further identifying molecular subtypes within or in addition to previously identified classes. The Consensus Cumulative Distribution Function (CDF) and Delta area plots graphically illustrate the optimal choice of cluster number. The CDF reaches an approximate maximum at k = 5 (Fig. S3).

**Figure 3:**
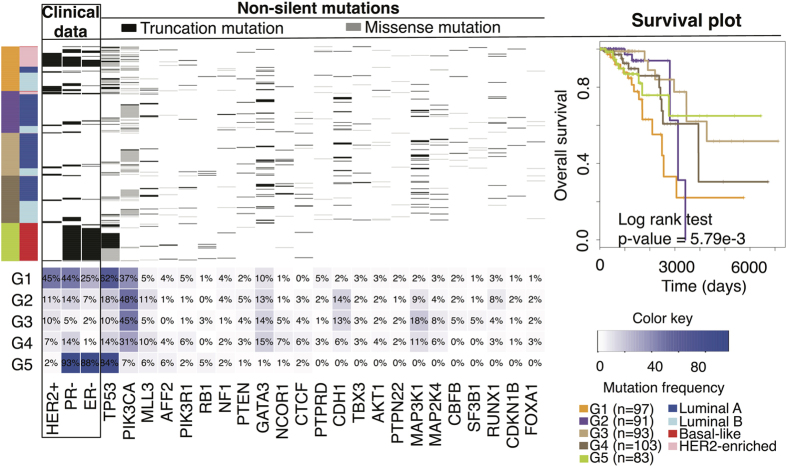
Breast cancer subtype discovery using the expression of glycosyltransferase genes. To provide a quantitative evidence for the prediction of a number of possible clusters within the TCGA breast cancer dataset, consensus clustering and a class discovery technique^48^ were conducted (See also Fig. S3A,B). Furthermore, to group samples into subtypes based on the expression of glycosyltransferase genes, a k-means clustering was performed and cluster significance was evaluated using ‘SigClust’ package^49^, and all class boundaries were statistically significant (See also Fig. S3C). To investigate whether the identified groups (using k-means clustering), specific to breast cancer may represent clinically distinct subgroups of patients, univariate survival analyses (comparing subtypes, k = 2 to k = 10, with respect to the overall survival) was performed (See also Fig. S3D). Tumour samples (n = 467) in TCGA Agilent Microarray dataset for BRCA are grouped in five subtypes: G1, n = 97; G2, n = 91; G3, n = 93; G4, n = 103; G5, n = 83. These are shown with clinical data and somatic mutation information for each group. A sample grouping based on the TCGA 2012 study^33^ is illustrated with a separate colour side bar. From left to right columns show sample’s clinical data and somatic mutation patterns for the significantly mutated genes (top) along with their frequencies in percentage (bottom). HER2+, PR-, ER- and truncation mutation were coloured in black, while wild type and missense mutations were coloured in white and grey respectively. The GT gene discovered subclasses are shown in the farthest left column and the corresponding clinical subclasses are matched to the groups G1–G5. The colour scheme for GT gene discovered subclasses and clinical used subclasses are shown on the farthest right. To the furthest right is a plot of the survival timelines for G1–G5 subtypes.

Evaluating the prognostic capabilities of breast cancer subtypes derived from the expression profile of GT genes we find that survival curves significantly differ between subtypes when breast cancer samples are divided into these subgroups (Fig. 3). According to the study carried out by Perou and colleagues (2000), Sorlie and colleagues (2001) and several others, the existence of four major breast cancer subtypes (Luminal-type A, Luminal-type B, Basal-like and HER2-enriched) has become a consensus in breast cancer classification^31^,^32^. Furthermore, a study carried out by The Cancer Genome Atlas Network^33^, provided key insights into these four previously defined breast cancer subtypes and discovered significant molecular heterogeneity in each subtypes.

The Luminal-type A, Luminal-type B, Basal-like and HER2-enriched breast cancer subtypes, discovered from tumour morphology and characterized by molecular taxonomy^34^ informs current patient therapy. Therefore, it is convenient to corroborate subclass discovery for breast cancer from GT gene expressions, against the more than 30 years of morphology studies of the above clinical subtypes. Our results show the most significant difference between breast cancer subtypes when data is divided into five groups (Log rank test p-value = 5.79e-3, Fig. 3). Moreover, the survival curves significantly differ between subtypes when divided into five groups (Figs 3 and S3).

Aligning the clinically applied molecular cancer diagnostics, somatic mutation patterns and the survival curves with the GT gene expression detected subtypes (G1–G5) results in informative correlation (Fig. 3). The G1 overlap with HER2-enriched and the G5 strongly overlap with basal-like subtypes, while luminal (A and B) are mainly distributed between the other three subtypes (G2, G3 and G4). The expression patterns for proteins that guide clinical treatment were significantly more conserved within G1 (HER2+: 45%, PR-: 44% and ER-: 25%, considered as HER2- enriched) and G5 (HER2+: 2%, PR-: 93% and ER-: 84%, considered as basal-like) compared with the other three subtypes (Fig. 3).

The samples in G1 and G5 subtypes are both mostly mutated for TP53 (62% and 84% respectively), while they are differently mutated for PIK3CA (37% and 7% respectively). This corresponds with the findings from the TCGA analysis of HER2-enriched and basal-like groups^35^. In addition, GATA3 mutations are barely noticeable in G5 (1%) compare with G1 to G4 (10% to 15%) as similarly observed in luminal subtypes or ER+ tumours in another study^35^ lending further support that G5 is basal-like and is segregated from other subtypes. Furthermore, the rate of GATA3 mutations in luminal subtypes (G2: 13%, G3: 14% and G4: 15%) is in line with the results of other studies^35^,^36^,^37^.

## Conclusion

The combined effect of differential expression leading to cancer segregation and highly ranked importance of GT genes in cancer identification emphasizes that the biochemical pathways underlying key phenotypes across cancers differ significantly. We have detailed a method of identification of cancer types using supervised algorithms that have been trained to evaluate quantified glycosyltransferase gene expression in a sample from the patient TCGA data. The method may be used in combination with a kit comprising glycosyltransferase gene capture probes or primers. In a clinical setting the method and/or kit may be used to monitor the treatment of cancer.

Furthermore we have shown that cancer subtype classification and/or identification of a cancer subtype within a specific cancer type is possible through the use of unsupervised and supervised algorithms that have been trained to evaluate quantified glycosyltransferase gene expression. In a clinical setting this could be used to select an appropriate treatment regime for a patient and/or to monitor the response of the patient to the cancer treatment. We have specifically demonstrated the discovery of subclasses in breast cancer. We chose breast cancer because of the extensive clinical data and clinically defined subclasses against which we could measure the GT expression discovered subclasses. Here we found that the mutation frequency of TP53 and PIK3CA combined with the protein expression mapping to G1 and G5 provides convincing evidence that these two subtypes identified from GT expression profiles correspond with HER2-enriched and basal-like subtypes respectively and are distinct from the luminal subtypes.

## Methods

### Data Analytics Procedures

#### Data preparation

Agilent Microarray data of 210 glycosyltransferase (GT) genes of 1893 patients was retrieved from TCGA (http://tcga-data.nci.nih.gov/tcga/) representing breast invasive carcinoma, ovarian serous cystadenocarcinoma, glioblastoma multiforme, kidney renal clear cell carcinoma, colon adenocarcinoma and lung squamous cell carcinoma. All the analyses have been done in R^38^. Batch effects were evaluated using ‘ComBat’ function and principal component analysis (PCA) in ‘sva’^39^ and ‘psych’^40^ packages.

Cancer segregation. Hierarchical clustering, using ‘cluster’ package^41^, and PCA were performed.

The TCGA pre-processed data (level 3) was used for all analysis. Pre-processing steps were not repeated in GSE20624 dataset due to the extensive similarity between TCGA and GSE20624 datasets for RNA extraction (Stratagene), labeling^42^, microarray platform (Agilent two-channel using UNC custom Microarrays) and pre-processing methods. The log2(R/G) of the gene expression was LOWESS normalized, row (gene) median centered, and column (sample) standardized. However, TCGA and GSE20624 breast cancer dataset were assessed using visualization methods such as boxplots with quintile normalization applied to the datasets before analysis.

#### Differential expression and survival analysis

Pairwise comparisons were employed using ‘limma’ package^43^ (q-value ≤ 0.005 and 2 fold change). The ‘decideTests’ function was used to assigning binary values (1:up-regulated, −1:down-regulated and 0:not detected) to the genes. A gene in a specific cancer is up-regulated if the median of all pairwise comparisons is 1 and it is down-regulated (−1) in none of the comparisons and vise versa. Correlation between patient survival and GT gene expression was estimated using ‘survival’ package^44^. Samples in the dataset were divided into two groups for each gene (0:expression value above median and 1:below median), and compared to each other in terms of overall outcome. We used the Log rank test (Mantel–Cox test) which was derived as the score test using the Cox proportional hazards model. Here it is implemented via the ‘coxph’ function in ‘survival’ package in R (see Supporting Information for further details). The Log rank test p-value is a measure that explains if the survival curves for various groups are significantly different (p < 0.05).

#### Classifier development

A GT gene classifier was developed using the ‘pamr’ package (http://cran.r-project.org/web/packages/pamr). The gene importance was estimated using the ‘caret’ package^45^. 10-fold cross validation, internal (randomly dividing dataset into 70% training and 30% test in 100 experiments) and external (GSE20624) tests were carried out. The accuracy measures derived from a confusion matrix, ROC curve and its confidence interval (CI) for internal test were estimated using ‘pROC’ package^46^.

#### Breast cancer subtyping

Consensus clustering^47^ was conducted using ‘ConsensusClusterPlus’ package^48^. Samples are grouped into five subtypes using k-means clustering in ‘cluster’ package^41^. Cluster significance was evaluated using ‘SigClust’ package^49^. Survival analyses (comparing k = 2 to k = 10) was performed using ‘survival’ package^44^.

## Additional Information

**How to cite this article**: Ashkani, J. and Naidoo, K. J. Glycosyltransferase Gene Expression Profiles Classify Cancer Types and Propose Prognostic Subtypes. *Sci. Rep*. **6**, 26451; doi: 10.1038/srep26451 (2016).

## Supplementary Material

Supplementary Information

## Figures and Tables

**Table 1:** Summary of 10-fold cross validation of GT gene classifier.

Cancer type	BRCA	COAD	GBM	KIRC	LUSC	OV	Class error rate
BRCA	521	0	0	0	4	6	0.019
COAD	0	152	0	0	1	1	0.013
GBM	0	0	402	0	0	1	0.002
KIRC	0	0	0	71	0	1	0.014
LUSC	0	0	1	0	149	5	0.039
OV	0	0	1	0	1	576	0.003
Overall error rate							0.012
