# Supplementary material for: Glycosyltransferase Gene Expression Profiles Classify Cancer Types and Propose Prognostic Subtypes
Source: Sci Rep. 2016 May 20;6:26451. doi: 10.1038/srep26451 (PMC4873817; doi:10.1038/srep26451)
Supplement: Supplementary Information [file srep26451-s1.pdf]

Glycosyltransferase Gene Expression Profiles Classify Cancer Types and Propose Prognostic Subtypes

Jahanshah Ashkani[1,2], Kevin J. Naidoo[1,2*]

[1] Scientific Computing Research Unit, Faculty of Science, University of Cape Town, Rondebosch, 7701.

[2] Department of Chemistry, Faculty of Science, University of Cape Town, Rondebosch, 7701.

* Corresponding Author: Kevin J. Naidoo (kevin.naidoo@uct.ac.za)

Supplementary Information: Methods Details and Data

Though next generation sequencing can produce more accurate data with higher sensitivity[1], we used microarray data because of the availability of a larger number of samples with adequate follow up information, which were retrieved from The Cancer Genome Atlas (TCGA) data portal (https://tcga-data.nci.nih.gov/tcga/) and also availability of an independent dataset, GSE20624[2], as external test for the developed classifier. The Cancer Genome Atlas (TCGA) Research Network has improved our understanding in cancer biology through profiling and analyzing large numbers of human tumors. The resulting rich data offer a great opportunity to improve a coherent picture of variation across tumors[3,4]. For the purpose of this study, Agilent Microarray (Agilent 244K custom gene expression) data of 1893 samples from published TCGA (http://tcga-data.nci.nih.gov/tcga/) representing six cancer types including breast: breast invasive carcinoma [BRCA, n=531], ovary: ovarian serous cystadenocarcinoma [OV, n=578], brain: glioblastoma multiforme [GBM, n=403], kidney: kidney renal clear cell carcinoma [KIRC, n=72], colon: colon adenocarcinoma [COAD, n=154] and lung: lung squamous cell carcinoma [LUSC, n=155]) were combined, while normal and control samples were excluded.

A list of human glycosyltransferase (GT) genes was retrieved through filtering several publicly available databases such as Kyoto Encyclopedia of Genes and Genomes database (KEGG/GENES) (http://www.genome.jp/kegg/genes.html), Carbohydrate-Active Enzymes database (CAZy) databases (http://www.cazy.org/), and literature search. KEGG/GENES is a pool of manually curated genes retrieved mainly from NCBI RefSeq[5-7]. Furthermore, CAZy provides an online and regularly updated access to family classification of CAZymes corresponding to proteins released in the daily releases of GenBank (ftp://ftp.ncbi.nih.gov/genbank/daily-nc)[8]. Supplementary Table 1 contains a list of human glycosyltransferase gene symbols with their Entrez numbers.

The expression dataset of glycosyltransferases was built through combining the TCGA expression datasets of six investigated cancer types (*i.e.* breast, brain, colon, kidney, lung and ovary) and further retrieving the expression of 210 glycosyltransferase genes from the combined dataset.

Batch effects are commonly observed systematic non-biological variation between groups of samples due to experimental artifacts, such as processing date, lab, or technician. Combining samples from multiple batches can cause the true biological variation in a high-throughput experiment to be obscured by variation due to batch[9]. However, the correlations of batch effects (technical and biological artifacts) with the outcome are common and critical to address[10], correcting for batch effects when there is no significant effects may result in removing biological variation instead of the systematic non-biological variation due to batch[9]. Therefore, a simple test was performed to evaluate existing of batch effects in combined dataset with comparing box plots, QQ-plots and applying a t-test analysis before and after using an Empirical Bayes batch effect correction method, *i.e.* ComBat, implemented in 'sva' package[11] in R[12]. The result of this analysis clearly illustrated that no significant batch effects in the dataset (t-test p-value=0.5, Supplementary Figure 1a) and also there is no specific color grouping observed based on the batch effects in the principal component loading plot for PC1 to PC3 in the combined dataset (Supplementary Figure 1b). However a significant grouping is observed while samples are colored based on the cancer type (Fig. 1a) confirming the batch effects do not stimulate cancer type grouping. In addition, separate principal component analyses were performed to investigate the batch effects in each TCGA cancer type while samples are colored based on the batch numbers and they have not shown any grouping based on the batch numbers in each cancer type (Supplementary Figure 1c).

To separate cancer types based on the expression of glycosyltransferase genes, a principal component analysis was performed using 'psych' package[13] in R. Furthermore, a hierarchical average linkage clustering performed on GT genes and cancer types across the complete 1893 sample set using 'cluster' package[13] in R. The result of this analysis reveals that the expression profile of GT genes not only separates six cancer types but also represents a unique molecular entity with similarity to lung cancer for basal-like samples (TNBC, n=83, colored in black in the TNBC sidebar in Supplementary Figure 2), which is in line with the result of Prat and colleagues (2013) investigating the expression of 3486 most variable genes across six different cancer types from TCGA data[3].

To better understand how the expression of glycosyltransferase genes contribute to separation of cancer types from each other and to investigate dominant glycan-specific changes occur in carcinogeneic process of each cancer type, the expression of glycosyltransferase genes was compared among the cancer types and the association of glycosyltransferase genes to patient survival was studied. For this purpose, differential expression analyses were carried out using 'limma' package[14] in R. Genes with q-value > 0.005 and 2 fold change were considered as a differentially expressed gene in

pairwise comparisons, while a 'decideTests' function in 'limma' package was used to assigning binary values (*i.e.* 1: up-regulated, -1: down-regulated and 0: not detected) to these genes. Finally, a gene in a specific cancer types was considered to be up-regulated if the median of all pairwise comparisons was 1 and it is down-regulated (-1) in none of the comparisons and wise versa (Supplementary Table 2). Correlation between patient survival and glycosyltransferase gene expression was performed using log rank test implemented in 'survival' package[15] in R, while samples in all cancer types were divided into two groups for each gene (0: samples that showed gene expression value above median and 1: below median), and then compared to each other in terms of overall outcome (Supplementary Table 2). In addition, Supplementary Table 3 shows the expression of glycosyltransferase genes between normal and malignant in various cancers in several studies.

Having established that the expression profile of glycosyltransferase genes are able to separate six cancers we explore the development of a GT gene classifier using shrunken centroid approach[16] in 'pamr' package (http://cran.r-project.org/web/packages/pamr) in R, which is able to identify cancer type from a random sample. Furthermore, 'caret' package[17] in R was used to rank the gene importance in a supervised learning model (pam model).

For the purpose of error estimation of training model (pam classifier) in the assignment of samples to the right cancer types, a 10-fold cross validation technique was carried out using 'pamr' package in R. In addition, internal and independent/external tests were carried out to evaluate the performance of the pam classifier using the expression of glycosyltransferase genes. For this purpose, the glycosyltranseferases' expression dataset was randomly split hundred times into training (70%) and test (30%) sets. Training sets were used to build a model, which were then applied to the testing sets. Finally, the median values were used to assign each sample to a specific cancer type. The result of this analysis was used for accuracy measurement calculation summarized in Supplementary Table 4. Given a classifier and a sample, there are four possible outcomes: true positive, true negative, false positive and false negative. It is true positive if the sample is positive and it is classified as positive and it is false negative if it is classified as negative. It is true negative if the sample is negative and it is classified as negative and it is a false positive if it is classified as positive. Given a classifier and a set of samples (the test set), a two-by-two confusion matrix (also called a contingency table) can be constructed representing the dispositions of the set of samples, see Fawcett (2006) for more information and equations[16]. Furthermore, the ROC (Receiver Operating Characteristics) curve has been extensively studied and applied in medical diagnosis since the 1970s[18,19] and the area under the ROC (AUC)[20] has become an important performance measure in this regard, since it is invariant to operating conditions[21]. The accuracy measures derived from a confusion matrix, the area under the receiver operating characteristic (ROC) curve and its confidence interval (CI) for internal test (Supplementary Table 4), clearly shows the potential of gene expression profiling of glycosyltransferase in tumor type identification/separation with high accuracy, sensitivity and specificity for all investigated cancer types.

Since training algorithms look for patterns in the training dataset, a classifier that relies on these spurious patterns will have higher accuracy on the training examples than it will on the whole population. Therefore, it is extremely critical to evaluate the performance of a classifier on an independent test set. For this purpose, training sets of previous test (internal test) have been used for an external (independent) test that examines 293 breast cancer samples existing in GPL1390 platform of GSE20624[2]. GSE20624 (GPL1390) data is not included in TCGA while it uses the same microarray platform with TCGA datasets, however, only 177 glycosyltransferase are common between training (TCGA based) and this dataset.

In terms of breast cancer subtyping, to provide a quantitative evidence for the prediction of a number of possible clusters within the TCGA breast cancer dataset, consensus clustering plus class discovery technique [24] was conducted using 'ConsensusClusterPlus' package[22] in R. Consensus clustering is a clustering framework that has been widely used for cancer subtyping. In this technique, the same clustering algorithm is applied multiple times to different subsets of the data and a consensus result is collected to better describe the similarities between samples[23]. The consensus Cumulative Distribution Function (CDF) and Delta area plots are the graphical representations to illustrate at what number of clusters, the CDF reaches an approximate maximum and at which k (number of groups) there is no significant increase in CDF curve, respectively. The result of consensus clustering analysis was graphically represented as heatmaps for the consensus matrices of k=2 to k=10. Accordingly, microarrays are placed in both rows and columns of the consensus matrices and consensus value ranges are colored by white to dark blue, indicating that samples never cluster together and always cluster together, respectively (Supplementary Figures 3a and b). Furthermore, to group samples into subtypes based on the expression of glycosyltransferase genes, a k-means clustering was performed using 'cluster' package in R. Cluster significance was evaluated using 'SigClust' package[24] in R, and all class

boundaries were statistically significant (Supplementary Figure 3c). To investigate whether the identified groups (using k-means clustering), specific to breast cancer may represent clinically distinct subgroups of patients, univariate survival analyses (comparing subtypes, k=2 to k=10, with respect to the overall survival) was performed (Supplementary Figure 3d) using 'survival' package in R, while previously identified normal-like[25], metastatic samples and the samples with missing survival information in the corresponding patient were excluded (n=467).

Supplementary Figures:

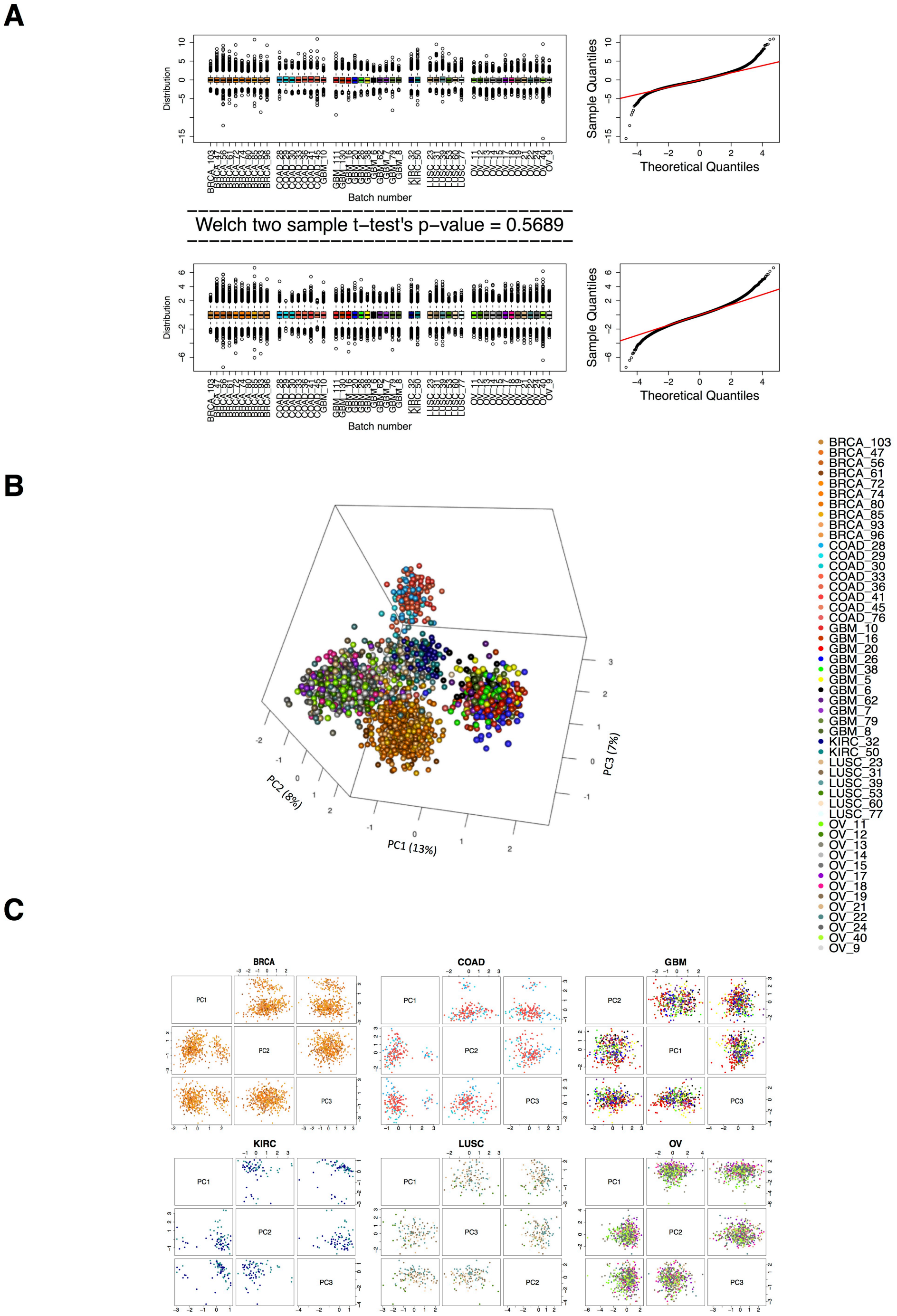

Supplementary Figure 1 | Evaluation of batch effects across combined and separated datasets. **a**, Comparison of sample distribution before and after applying a batch correction method, ComBat[11]. Box plots and QQ-plots illustrate the distribution of samples in dataset before (top) and after (down) applying ComBat and a t-test compares these two datasets. **b**, Visualization of batch effects across combined dataset. Principal component loading plot for PC1-PC3, while samples colored based on the batch numbers. **c**, Evaluation of batch effects within each cancer type. Pairwise principal component loading plots for PC1-PC3, while samples are colored based on the batch numbers in each cancer type (*i.e.* breast: breast invasive carcinoma [BRCA, n=531], ovary: ovarian serous cystadenocarcinoma [OV, n=578], brain: glioblastoma multiforme [GBM, n=403], kidney: kidney renal clear cell carcinoma [KIRC, n=72], colon: colon adenocarcinoma [COAD, n=154] and lung: lung squamous cell carcinoma [LUSC, n=155]). The numbers after '_' character represent the batch numbers.

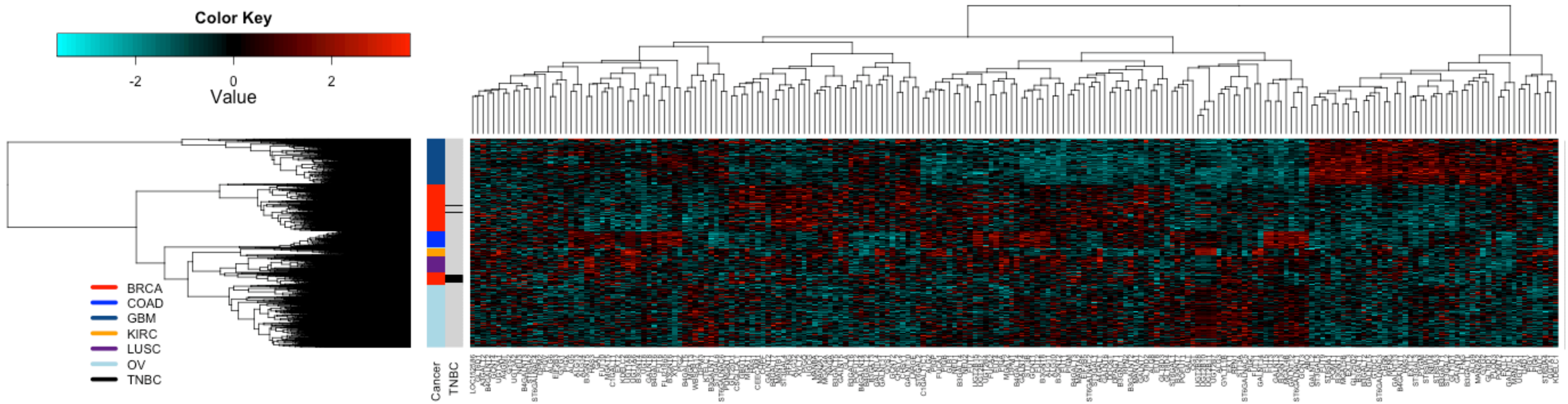

Supplementary Figure 2 | The expression profile of Glycosyltransferases across six various cancer types. Samples are colored based on the cancer type in Cancer side bar (*i.e.* breast: breast invasive carcinoma [BRCA, n=531], ovary: ovarian serous cystadenocarcinoma [OV, n=578], brain: glioblastoma multiforme [GBM, n=403], kidney: kidney renal clear cell carcinoma [KIRC, n=72], colon: colon adenocarcinoma [COAD, n=154] and lung: lung squamous cell carcinoma [LUSC, n=155]). Breast cancer clustered into two distinguished groups, which one of them (smaller one) contains most of the triple negative breast cancer samples (TNBC, n=83), which colored in black in the TNBC sidebar.

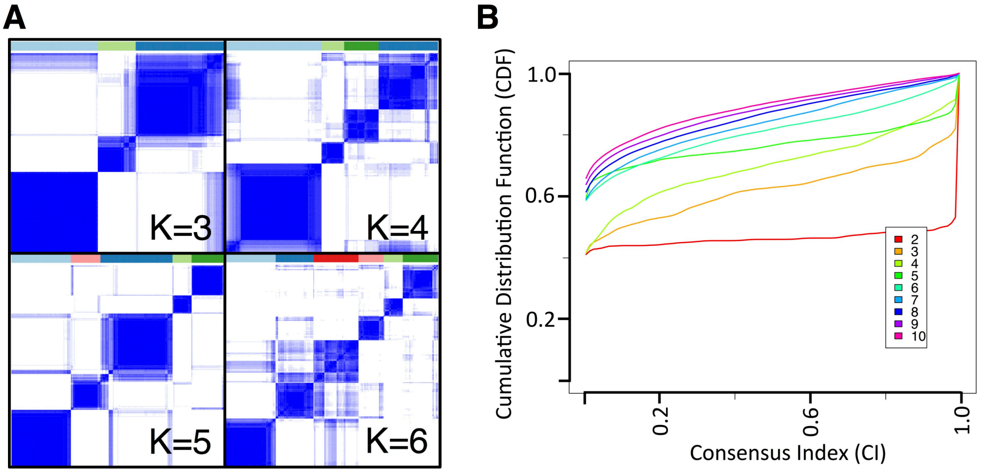

C

Groups	G1	G2	G3	G4
G1				
G2	2.50E-05			
G3	6.51E-04	9.71E-04		
G4	3.98E-06	3.78E-06	1.36E-03	
G5	1.03E-14	6.80E-14	4.51E-13	8.93E-17

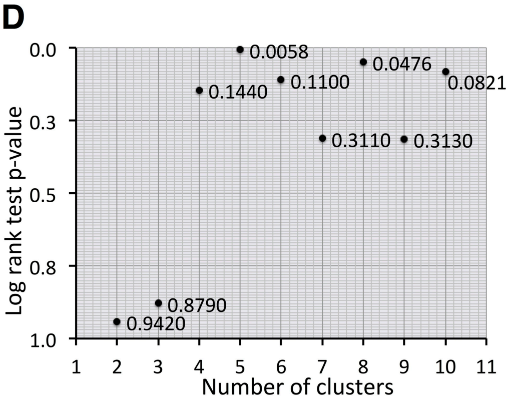

Supplementary Figure 3 | Identification of breast cancer subtypes using glycosyltransferase expression profile.

a, Consensus clustering matrix of 467 breast cancer TCGA samples for k=3 to k=6. **b**, Consensus CDF for k=2 to k=10. **c**, SigClust p-values for all pairwise comparisons of five clusters/groups. **d**, Log rank test p-values investigating the relation of number of subtypes (k=2 to k=10) with survival information.

Supplementary Tables:

Supplementary Table 1 | List of 210 glycosyltransferase genes presented in TCGA Agilent microarray expression datasets.

Gene symbol	Entrez ID.	Gene symbol	Entrez ID.	Gene symbol	Entrez ID.
A3GALT2	127550	FUT11	170384	MGAT3	4248
A4GALT	53947	FUT2	2524	MGAT4A	11320
A4GNT	51146	FUT3	2525	MGAT4B	11282
ABO	28	FUT4	2526	MGAT5	4249
ALG10	84920	FUT5	2527	MGAT5B	146664
ALG11	440138	FUT6	2528	NAGA	4668
ALG12	79087	FUT7	2529	NEU1	4758
ALG13	55849	FUT8	2530	NEU2	4759
ALG14	199857	FUT9	10690	NEU3	10825
ALG2	85365	GAL	51083	NEU4	129807
ALG3	10195	GALC	2581	OGT	8473
ALG5	29880	GALNT10	55568	PIGA	5277
ALG6	29929	GALNT11	63917	PIGB	9488
ALG8	79053	GALNT12	79695	PIGC	5279
ALG9	79796	GALNT13	114805	PIGH	5283
B3GALNT1	8706	GALNT14	79623	PIGM	93183
B3GALNT2	148789	GALNT2	2590	PIGP	51227
B3GALT1	8708	GALNT3	2591	PIGQ	9091
B3GALT2	8707	GALNT4	8693	PIGV	55650
B3GALT4	8705	GALNT5	11227	PIGX	54965
B3GALT5	10317	GALNT6	11226	PIGY	84992
B3GALT6	126792	GALNT7	51809	PIGZ	80235
B3GALTL	145173	GALNT8	26290	PLOD3	8985
B3GAT1	27087	GALNT9	50614	POFUT1	23509
B3GAT3	26229	GALNTL1	57452	POFUT2	23275
B3GNT1	11041	GALNTL2	117248	POMGNT1	55624
B3GNT2	10678	GALNTL4	374378	POMT1	10585
B3GNT3	10331	GALNTL5	168391	POMT2	29954
B3GNT4	79369	GALT	2592	PYGM	5837
B3GNT5	84002	GBGT1	26301	RFNG	5986
B3GNT6	192134	GCNT1	2650	RPN1	6184
B3GNT7	93010	GCNT2	2651	RPN2	6185
B3GNT8	374907	GCNT3	9245	ST3GAL1	6482
B3GNTL1	146712	GCNT4	51301	ST3GAL2	6483
B4GALNT1	2583	GGTA1	2681	ST3GAL3	6487
B4GALNT2	124872	GLA	2717	ST3GAL4	6484
B4GALNT3	283358	GLB1	2720	ST3GAL5	8869
B4GALNT4	338707	GLT1D1	144423	ST3GAL6	10402
B4GALT2	8704	GLT25D1	79709	ST6GAL1	6480
B4GALT3	8703	GLT25D2	23127	ST6GAL2	84620
B4GALT4	8702	GLT6D1	360203	ST6GALNAC1	55808
B4GALT5	9334	GLT8D1	55830	ST6GALNAC2	10610
B4GALT6	9331	GLT8D2	83468	ST6GALNAC3	256435
B4GALT7	11285	GLT8D3	283464	ST6GALNAC4	27090
C1GALT1	56913	GLT8D4/GXYLT2	727936	ST6GALNAC5	81849
C1GALT1C1	29071	GTDC1	79712	ST6GALNAC6	30815
CEECAM1	51148	GYG1	2992	ST8SIA1	6489
CHGN/GALNACT1	55790	GYG2	8908	ST8SIA2	8128
CHPF	79586	GYLTL1B	120071	ST8SIA3	51046
CHSY.2	337876	GYS1	2997	ST8SIA4	7903
CHSY1	22856	GYS2	2998	ST8SIA5	29906
CSGLCA.T	54480	HAS1	3036	STT3A	3703
DAD1	1603	HAS2	3037	STT3B	201595
DDOST	1650	HAS3	3038	UGCG	7357
DPAGT1	1798	KDELC1	79070	UGT1A6	54578

DPM1	8813	KDELC2	143888	UGT1A8	54576
DPM2	8818	KTELC1	56983	UGT2A1	10941
DPM3	54344	LARGE	9215	UGT2B10	7365
EIF2B3	8891	LFNG	3955	UGT2B11	10720
EIF2B5	8893	LOC152586	152586	UGT2B15	7366
EXT1	2131	MAN1A1	4121	UGT2B17	7367
EXT2	2132	MAN1A2	10905	UGT2B28	54490
EXTL1	2134	MAN1B1	11253	UGT2B4	7363
EXTL2	2135	MAN1C1	57134	UGT2B7	7364
EXTL3	2137	MAN2A1	4124	UGT3A1	133688
FLJ21865	64772	MAN2A2	4122	UGT3A2	167127
FUCA1	2517	MANBA	4126	UGT8	7368
FUCA2	2519	MFNG	4242	WBSCR17	64409
FUT1	2523	MGAT1	4245	XYLT1	64131
FUT10	84750	MGAT2	4247	XYLT2	64132

Supplementary Table 2 | List of differentially expressed glycosyltransferase genes in six cancer types compare to each other along with log rank test p-value of each gene in survival analysis. BRCA: breast cancer; COAD: colon cancer; GBM: brain; KIRC: kidney; LUSC: lung; OV: ovarian; ↑: up-regulated; ↓: down-regulated; □: not detected.

Glycan structure abberation	Gene symbol	Cancer type						Log rank test p-value
		BRCA	COAD	GBM	KIRC	LUSC	OV	
N-glycans								
Precursor synthesis								
	ALG10	□	↑	↓	□	↑	↑	5.11E-15
	ALG11	□	↑	↓	□	↑	↓	0.00E+00
	ALG12	↑	□	↓	□	□	↓	0.00E+00
	ALG3	□	↑	↓	↓	↑	↑	1.22E-06
	ALG5	↓	↑	□	□	□	↓	8.01E-01
Branching								
	MGAT4B	↓	↑	↓	□	↓	□	5.28E-08
	MGAT5B	↓	↓	↑	↓	↓	↓	0.00E+00
Biosecting								
	MGAT3	↓	↓	↑	□	↓	↑	0.00E+00
Core fucosylation								
	FUT8	↑	□	□	↓	↑	↑	7.27E-06
Increased α2,6-sialylation	ST6GAL1	↓	↑	↓	□	↓	□	2.77E-01
O-glycans								
Tn								
	GALNT10	□	□	□	□	□	↓	7.65E-01
	GALNT12	↓	↑	↓	□	□	□	7.96E-02
	GALNT13	↓	↓	↑	↓	□	□	0.00E+00
	GALNT14	□	↓	□	↓	□	□	2.26E-05
	GALNT3	□	↑	↓	↓	↑	↑	6.66E-15
	GALNT4	↓	↑	↓	↓	↓	□	8.29E-13
	GALNT5	□	↑	□	↓	□	↓	7.82E-01
	GALNT6	↑	↑	↓	↓	□	↑	0.00E+00
	GALNT7	↑	↑	↓	↓	□	↓	0.00E+00
	GALNT8	↓	↑	↓	↓	↓	↓	0.00E+00

	GALNT9	↓	↓	□	↑	↓	↓	0.00E+00
	GALNTL1	↑	↓	↑	□	↓	□	1.55E-03
	GALNTL2	↓	↓	↑	□	↓	↓	5.27E-13
	GALNTL4	↓	↓	□	↑	↑	↓	7.18E-01
	GALNTL5	↓	↓	↑	↓	↓	↓	0.00E+00
SialylTn (sTn)								
	ST6GALNAC1	↓	↑	□	↓	□	□	7.93E-01
	ST6GALNAC2	↑	↓	↓	↓	↑	↑	0.00E+00
	ST6GALNAC3	↓	↓	↑	↑	↓	↓	0.00E+00
	ST6GALNAC5	↑	□	□	↓	↑	↑	6.08E-02
	ST6GALNAC6	□	↓	↑	□	□	□	2.94E-13
SialylT (sT)								
	ST3GAL1	↑	↓	↓	↑	↑	↑	0.00E+00
Lewis antigens								
Sialyl Le(a)								
	ST3GAL3	↓	↓	↑	↑	↑	□	0.00E+00
	B3GALT1	↓	□	↑	↓	↓	□	0.00E+00
	B3GALT2	↓	↓	↑	↓	↓	↓	0.00E+00
	B3GALT5	□	↑	↓	□	↓	↓	5.02E-07
	B3GALTL	↓	□	↑	□	□	↓	1.15E-10
	FUT3	□	↑	↓	□	□	□	0.00E+00
Sialyl Le(x)								
	ST3GAL3	↓	↓	↑	↑	↑	□	0.00E+00
	ST3GAL6	↓	↓	↑	↑	□	↑	0.00E+00
	B4GALT4	↓	□	↓	↓	↑	□	8.51E-01
	B4GALT6	↓	↑	□	□	□	□	1.47E-07
	FUT3	□	↑	↓	□	□	□	0.00E+00
	FUT4	↓	↑	↓	↓	↓	↓	1.91E-07
	FUT5	↓	↑	↓	□	□	↓	3.37E-05
	FUT6	↓	↑	□	↑	↓	↓	7.62E-07
Glycosphingolipids								
Polysialic acid (PSA) linked to NCAM/SSEA-4								
	ST8SIA1	□	↓	↑	↓	□	↓	0.00E+00
	ST8SIA3	↓	↓	↑	↓	↓	↓	0.00E+00
	ST8SIA4	□	↓	□	↑	□	□	3.52E-06
	ST8SIA5	↓	↓	↑	↓	↓	□	0.00E+00
	A4GALT	□	↓	↓	↑	↑	↓	0.00E+00
Gb3								
	ST3GAL5	□	↓	↑	□	□	↓	0.00E+00
	B4GALNT1	↓	↓	↑	↓	↑	↓	0.00E+00
	FUT1	↑	↑	↓	□	↑	↑	0.00E+00
	FUT2	□	↑	↓	↓	□	□	0.00E+00

Supplementary Table 3 | Cell surface glycan changes and glycosyltransferase regulation between normal and malignant in various cancers. ↑: up-regulated; ↓: down-regulated.

Glycan structure aberration	Gene symbol	Expression in cancer	Reference(s)
N-glycans			
Precursor synthesis			
	ALG10	breast↑	[26]
	ALG3	breast↑	[26]
Branching			
	MGAT5B	breast↑	[26]
Biosecting			
	MGAT3	breast↓,colon↑,lung↑,kidney↑,ovarian↑	[26-32]
Core fucosylation			
	FUT8	breast↑, colon↑,ovarian↑	[26,31-33]
Increased α2,6-sialylation	ST6GAL1	breast↓,colon↑,brain↑,lung↑,ovarian↑	[29,34-38]
O-glycans			
Tn			
	GALNT10	breast↑	[26,39]
	GALNT12	breast↓	[26]
	GALNT14	lung↑	[40]
	GALNT3	breast↑,colon↑	[26,39,41]
	GALNT4	breast↑,kidney↑	[26,42]
	GALNT5	breast↑	[26,39]
	GALNT6	breast↑,ovarian↑	[26,39,43]
	GALNT7	breast↑	[26,39]
SialylTn (sTn)			
	ST6GALNAC2	breast↑	[39]
	ST6GALNAC3	breast↓	[26]
	ST6GALNAC5	brain↓	[36]
	ST6GALNAC6	breast↓,brain↓	[26,36]
SialylT (sT)			
	ST3GAL1	breast↑,colon↑,ovarian↑	[26,38,44-48]
Lewis antigens			
Sialyl Le(a)			
	ST3GAL3	breast↓,ovarian↓,colon↑	[26,38,39,49]
	B3GALT2	lung↓	[50]
	FUT3	breast↑,colon↓↑,brain↑,lung↑,ovarian↑	[29,36,39,51-57]
Sialyl Le(x)			
	ST3GAL3	breast↓,ovarian↓,colon↑	[26,38,39,49]
	ST3GAL6	breast↓↑,brain↓,ovarian↓	[26,29,38,44,55,58]
	FUT3	breast↑,colon↓↑,brain↑,lung↑,ovarian↑	[36,39,51-57]
	FUT4	breast↓↑,colon↑,lung↑,ovarian↑	[26,29,39,47,52,55,56,59,60]
	FUT5	breast↑, ovarian↑	[39,55,56]
	FUT6	colon↑, ovarian↑	[47,52,56,60-62]
Glycosphingolipids			

Polysialic acid (PSA) linked to NCAM/SSEA-4			
	ST8SIA1	breast↓↑, brain↑	26,36,39,63
Gb3			
	A4GALT	brain↓, lung↑	64,65
Fucosyl GM1-3/GD2,3			
	ST3GAL5	ovarian↓↑	38,66
	B4GALNT1	brain↑	67
	FUT1	breast↑,colon↑,ovarian↑	47,52,60,68-70
	FUT2	breast↑,lung↑,ovarian↑	71-73

Supplementary Table 4 | Accuracy measurements for the classifier using result of internal test. Accuracy measures derived from a confusion matrix, the area under the receiver operating characteristic (ROC) curve and its confidence interval (CI).

Measurement	Cancer type					
	BRCA	COAD	GBM	KIRC	LUSC	OV
Overall diagnostic power	0.71	0.91	0.78	0.96	0.91	0.69
Correct classification rate	0.98	0.99	0.99	0.99	0.99	0.99
Sensitivity	0.95	0.98	0.99	0.94	0.93	0.98
Specificity	0.99	0.98	0.99	0.99	0.99	0.99
False positive rate	1.5e-3	6.0e-4	1.3e-3	5.0e-4	2.3e-3	5.3e-3
False negative rate	4.7e-2	1.3e-2	7.4e-3	5.5e-2	6.4e-2	1.3e-2
Positive predictive power	0.99	0.99	0.99	0.98	0.97	0.98
Negative predictive power	0.98	0.99	0.99	0.99	0.99	0.99
Misclassification rate	1.4e-2	1.6e-3	2.6e-3	2.6e-3	7.4e-3	7.9e-3
Kappa	0.96	0.98	0.99	0.96	0.94	0.98
NMI n(s)	0.87	0.95	0.96	0.88	0.85	0.92
Area under the curve (%)	99.99	99.87	99.97	100	99.88	99.94
95% CI	99.98-100	99.62-100	99.90-100	99.99-100	99.75-100	99.87-100

*Diagnostic Power (DP) is determined by both its sensitivity and its specificity and it is the proportion correctly classified[74].
BRCA: breast invasive carcinoma (n=531), COAD: colon adenocarcinoma (n=154), GBM: glioblastoma multiforme (n=403), KIRC: kidney renal clear cell carcinoma (n=72), LUSC: lung squamous cell carcinoma (n=155) and OV: ovarian serous cystadenocarcinoma (n=578).

References:

1 Kim, H. & Bredel, M. Feature selection and survival modeling in The Cancer Genome Atlas. *Int J Nanomedicine* **8 Suppl 1**, 57-62, (2013).

2 Anders, C. K. *et al.* Breast carcinomas arising at a young age: unique biology or a surrogate for aggressive intrinsic subtypes? *J Clin Oncol* **29**, e18-e20, (2011).

3 Prat, A. *et al.* Genomic analyses across six cancer types identify basal-like breast cancer as a unique molecular entity. *Sci Rep* **3**, 3544, (2013).

4 Cancer Genome Atlas Research, N. *et al.* The Cancer Genome Atlas Pan-Cancer analysis project. *Nat Genet* **45**, 1113-1120, (2013).

5 Pruitt, K. D., Tatusova, T. & Maglott, D. R. NCBI Reference Sequence (RefSeq): a curated non-redundant sequence database of genomes, transcripts and proteins. *Nucleic Acids Res* **33**, D501-504, (2005).

6 Pruitt, K. D., Tatusova, T. & Maglott, D. R. NCBI reference sequences (RefSeq): a curated non-redundant sequence database of genomes, transcripts and proteins. *Nucleic Acids Res* **35**, D61-65, (2007).

7 Kanehisa, M. *et al.* From genomics to chemical genomics: new developments in KEGG. *Nucleic Acids Res* **34**, D354-357, (2006).

8 Lombard, V., Golaconda Ramulu, H., Drula, E., Coutinho, P. M. & Henrissat, B. The carbohydrate-active enzymes database (CAZy) in 2013. *Nucleic Acids Res* **42**, D490-495, (2014).

9 Reese, S. E. *et al.* A new statistic for identifying batch effects in high-throughput genomic data that uses guided principal component analysis. *Bioinformatics* **29**, 2877-2883, (2013).

10 Leek, J. T. *et al.* Tackling the widespread and critical impact of batch effects in high-throughput data. *Nat Rev Genet* **11**, 733-739, (2010).

11 Leek, J. T., Johnson, W. E., Parker, H. S., Jaffe, A. E. & Storey, J. D. The sva package for removing batch effects and other unwanted variation in high-throughput experiments. *Bioinformatics* **28**, 882-883, (2012).

12 Team, R. C. (2013). R: A language and environment for statistical computing. R Foundation for Statistical Computing. R Foundation for Statistical Computing, Vienna, Austria. URL http://www.R-project.org/

13 Maechler, M. *et al.* (2015). Cluster: Cluster Analysis Basics and Extensions. R Foundation for Statistical Computing, Vienna, Austria. URL https://cran.r-project.org/package=cluster

14 Ritchie, M. E. *et al.* limma powers differential expression analyses for RNA-sequencing and microarray studies. *Nucleic Acids Res*, gkv007, (2015).

15 Therneau, T. (2013). A package for survival analysis in S. R Foundation for Statistical Computing, Vienna, Austria. URL https://cran.r-project.org/package=survival

16 Fawcett, T. An introduction to ROC analysis. *Pattern Recogn Lett* **27**, 861-874, (2006).

17 Kuhn, M. (2012). The caret package. R Foundation for Statistical Computing, Vienna, Austria. URL https://cran.r-project.org/package=caret

18 Huang, J. & Ling, C. X. Using AUC and accuracy in evaluating learning algorithms. *IEEE T Knowl Data EN* **17**, 299-310, (2005).

19 Metz, C. E. in *Seminars in nuclear medicine.* 283-298 (Elsevier).

20 Bradley, A. P. The use of the area under the ROC curve in the evaluation of machine learning algorithms. *Pattern Recognit* **30**, 1145-1159, (1997).

21 Landgrebe, T. & Duin, R. P. in *Structural, Syntactic, and Statistical Pattern Recognition* 512-521 (Springer, 2006).

22 Monti, S., Tamayo, P., Mesirov, J. & Golub, T. Consensus clustering: a resampling-based method for class discovery and visualization of gene expression microarray data. *Machine learning* **52**, 91-118, (2003).

23 Verhaak, R. G. *et al.* Integrated genomic analysis identifies clinically relevant subtypes of glioblastoma characterized by abnormalities in PDGFRA, IDH1, EGFR, and NF1. *Cancer Cell* **17**, 98-110, (2010).
24 Huang, H., Liu, Y. & Marron, J. (2012). sigclust: Statistical Significance of Clustering. R Foundation for Statistical Computing, Vienna, Austria. URL https://cran.r-project.org/package=sigclust
25 TCGA. Comprehensive molecular portraits of human breast tumours. *Nature* **490**, 61-70, (2012).
26 Potapenko, I. *et al.* 398 Glycan gene expression signatures distinguish normal and malignant breast tissue; possible role in diagnosis and progression. *EJC Supplements* **8**, 102, (2010).
27 Shimodaira, K. *et al.* Carcinoma-associated Expression of Core 2 β-1,6-N-Acetylglucosaminyltransferase Gene in Human Colorectal Cancer: Role of O-Glycans in Tumor Progression. *Cancer Res* **57**, 5201-5206, (1997).
28 Chen, G. *et al.* Proteomic Analysis of Lung Adenocarcinoma: Identification of a Highly Expressed Set of Proteins in Tumors. *Clin Cancer Res* **8**, 2298-2305, (2002).
29 Petretti, T., Kemmner, W., Schulze, B. & Schlag, P. M. Altered mRNA expression of glycosyltransferases in human colorectal carcinomas and liver metastases. *Gut* **46**, 359-366, (2000).
30 Aoyagi, Y. *et al.* Alpha-Fetoprotein-Producing Renal Cell Carcinoma with Increased Activity of N-Acetylglucosaminyltransferase III. *Nephron* **74**, 409-409, (1996).
31 Abbott, K. L. *et al.* Focused glycomic analysis of the N-linked glycan biosynthetic pathway in ovarian cancer. *Proteomics* **8**, 3210-3220, (2008).
32 Yamashita, H. *et al.* Alteration in the metastatic potential of ovarian cancer cells by transfection of the antisense gene of β-1, 4-galactosyltransferase. *Oncol Rep* **10**, 1857-1862, (2003).
33 Muinelo-Romay, L., Villar-Portela, S., Cuevas Alvarez, E., Gil-Martín, E. & Fernández-Briera, A. α(1,6)Fucosyltransferase expression is an independent prognostic factor for disease-free survival in colorectal carcinoma. *Hum Pathol* **42**, 1740-1750, (2011).
34 Recchi, M.-A. *et al.* Multiplex Reverse Transcription Polymerase Chain Reaction Assessment of Sialyltransferase Expression in Human Breast Cancer. *Cancer Res* **58**, 4066-4070, (1998).
35 Dall'Olio, F., Chiricolo, M. & Lau, J. T. Y. Differential expression of the hepatic transcript of β-galactoside α2,6-sialyltransferase in human colon cancer cell lines. *Int J Cancer* **81**, 243-247, (1999).
36 Kroes, R. A., Dawson, G. & Moskal, J. R. Focused microarray analysis of glyco-gene expression in human glioblastomas. *J Neurochem* **103**, 14-24, (2007).
37 Wang, P.-H. *et al.* Enhanced expression of α 2,6-sialyltransferase ST6Gal I in cervical squamous cell carcinoma. *Gynecol Oncol* **89**, 395-401, (2003).
38 Wang, P.-H. *et al.* Altered mRNA expressions of sialyltransferases in ovarian cancers. *Gynecol Oncol* **99**, 631-639, (2005).
39 Carcel-Trullols, J. *et al.* Characterization of the glycosylation profile of the human breast cancer cell line, MDA-231, and a bone colonizing variant. *Int J Oncol* **28**, 1173-1183, (2006).
40 Wagner, K. W. *et al.* Death-receptor O-glycosylation controls tumor-cell sensitivity to the proapoptotic ligand Apo2L/TRAIL. *Nat Med* **13**, 1070-1077, (2007).
41 Schneider, F. *et al.* Overexpression of Sialyltransferase CMP-Sialic Acid:Galβ1,3GalNAc-R α6-Sialyltransferase Is Related to Poor Patient Survival inHuman Colorectal Carcinomas. *Cancer Res* **61**, 4605-4611, (2001).
42 Aoki, H. *et al.* Inhibition of motility and invasiveness of renal cell carcinoma induced by short interfering RNA transfection of β1,4GalNAc transferase. *FEBS Letters* **567**, 203-208, (2004).

43 Ryuko, K., Iwanari, O., Nakayama, S., Iida, K. & Kitao, M. Clinical evaluation of serum sialosyl-Tn antigen levels in comparison with CA 125 levels in gynecologic cancers. *Cancer* **69**, 2368-2378, (1992).
44 Burchell, J. *et al.* An α2,3 sialyltransferase (ST3Gal I) is elevated in primary breast carcinomas. *Glycobiology* **9**, 1307-1311, (1999).
45 Dalziel, M. *et al.* The Relative Activities of the C2GnT1 and ST3Gal-I Glycosyltransferases Determine O-Glycan Structure and Expression of a Tumor-associated Epitope on MUC1. *J Bio Chem* **276**, 11007-11015, (2001).
46 Brockhausen, I., Yang, J.-M., Burchell, J., Whitehouse, C. & Taylor-Papadimitriou, J. Mechanisms Underlying Aberrant Glycosylation of MUC1 Mucin in Breast Cancer Cells. *Eur J Biochem* **233**, 607-617, (1995).
47 Ito, H. *et al.* Altered mRNA expression of specific molecular species of fucosyl- and sialyl-transferases in human colorectal cancer tissues. *Int J Cancer* **71**, 556-564, (1997).
48 Hiraiwa, N., Ito, H., Zenita, K. & Kannagi, R. Structures, synthesis and functions of sialyl Le (a)/sialyl Le (x) antigens. *Nihon Rinsho* **53**, 1729-1734, (1995).
49 Dallolio, F., Chiricolo, M., Lollini, P. & Lau, J. T. Y. Human Colon Cancer Cell Lines Permanently Expressing α2,6-Sialylated Sugar Chains by Transfection with Rat β-Galactoside α2,6 Sialyltransferase cDNA. *Biochem Biophys Res Commun* **211**, 554-561, (1995).
50 Audfray, A. *et al.* A Recombinant Fungal Lectin for Labeling Truncated Glycans on Human Cancer Cells. *PLoS One* **10**, e0128190, (2015).
51 Hanski, C. *et al.* Fucosyltransferase III and sialyl-Lex expression correlate in cultured colon carcinoma cells but not in colon carcinoma tissue. *Glycoconj J* **13**, 727-733, (1996).
52 Kudo, T. *et al.* Up-regulation of a set of glycosyltransferase genes in human colorectal cancer. *Lab Invest* **78**, 797-811, (1998).
53 Nishihara, S. *et al.* Molecular mechanisms of expression of Lewis b antigen and other Type I Lewis antigens in human colorectal cancer. *Glycobiology* **9**, 607-616, (1999).
54 Opolski, A. *et al.* Metastatic potential of human CX-1 colon adenocarcinoma cells is dependent on the expression of sialosyl Le a antigen. *Clin Exp Metastas* **16**, 673-681, (1998).
55 Julien, S. *et al.* Selectin ligand sialyl-Lewis x antigen drives metastasis of hormone-dependent breast cancers. *Cancer Res* **71**, 7683-7693, (2011).
56 Escrevente, C. *et al.* Different expression levels of α3/4 fucosyltransferases and Lewis determinants in ovarian carcinoma tissues and cell lines. *Int J Oncol* **29**, 557-566, (2006).
57 Togayachi, A. *et al.* Up-regulation of Lewis enzyme (Fuc-TIII) and plasma-type α1,3Fucosyltransferase (Fuc-TVI) expression determines the augmented expression of sialyl Lewis x antigen in non-small cell lung cancer. *Int J Cancer* **83**, 70-79, (1999).
58 Reboul, P., George, P., Geoffroy, J., Louisot, P. & Broquet, P. Study of o-glycan sialylation in c6 cultured glioma cells: Regulation of a β-galactoside α2,3 sialyltransferase activity by ca2+/calmodulin antagonists and phosphatase inhibitors. *Biochem Biophys Res Commun* **186**, 1575-1581, (1992).
59 Gu, H., Ni, C. & Zhan, R. The expression of CD15 mRNA CD44v6 mRNA and nm23H1 mRNA in breast cancer and their clinical significance. *Zhonghua yi xue za zhi* **80**, 854-857, (2000).
60 Trinchera, M. *et al.* The biosynthesis of the selectin-ligand sialyl Lewis x in colorectal cancer tissues is regulated by fucosyltransferase VI and can be inhibited by an RNA interference-based approach. *Int J Biochem Cell Biol* **43**, 130-139, (2011).
61 Hiller, K. M. *et al.* Transfection of α(1,3)fucosyltransferase antisense sequences impairs the proliferative and tumorigenic ability of human colon carcinoma cells. *Mol Carcinog* **27**, 280-288, (2000).

62 Osumi, D. *et al.* Core fucosylation of E‐cadherin enhances cell–cell adhesion in human colon carcinoma WiDr cells. *Cancer Sci* **100**, 888-895, (2009).
63 Nakamura, O., Iwamori, M., Matsutani, M. & Takakura, K. Ganglioside GD3 shedding by human gliomas. *Acta neurochirurgica* **109**, 34-36, (1991).
64 Dhandapani, K. M., Mahesh, V. B. & Brann, D. W. Curcumin suppresses growth and chemoresistance of human glioblastoma cells via AP-1 and NFκB transcription factors. *J Neurochem* **102**, 522-538, (2007).
65 Oremek, G., Kukshaĭte, R., Sapoutzis, N. & Ziolkovski, P. The significance of TU M2-PK tumor marker for lung cancer diagnostics. *Klin Med* **85**, 56-58, (2006).
66 Prinetti, A. *et al.* A Glycosphingolipid/Caveolin-1 Signaling Complex Inhibits Motility of Human Ovarian Carcinoma Cells. *J Bio Chem* **286**, 40900-40910, (2011).
67 Oblinger, J. L. *et al.* Diagnostic and prognostic value of glycosyltransferase mRNA in glioblastoma multiforme patients. *Neuropathol Appl Neurobiol* **32**, 410-418, (2006).
68 Katano, M. Hedgehog signaling pathway as a therapeutic target in breast cancer. *Cancer Lett* **227**, 99-104, (2005).
69 Sun, J. *et al.* Elevated expression of H type GDP-L-fucose:beta-D-galactoside alpha-2-L-fucosyltransferase is associated with human colon adenocarcinoma progression. *Proc Natl Acad Sci* **92**, 5724-5728, (1995).
70 Liu, J.-J. *et al.* Lewis (y) antigen stimulates the growth of ovarian cancer cells via regulation of the epidermal growth factor receptor pathway. *Oncol Rep* **23**, 833-841, (2010).
71 Liu, L. *et al.* The identification and characterization of novel N-glycan-based biomarkers in gastric cancer. *PLoS One* **8**, e77821, (2013).
72 Hebbar, M. *et al.* Prognostic value of tumoral sialyltransferase expression and circulating E-selectin concentrations in node-negative breast cancer patients. *Int J Biol Markers* **18**, 116-122, (2003).
73 Roselli, M. *et al.* Soluble selectin levels in patients with lung cancer. *Int J Biol Markers* **17**, 56-62, (2002).
74 Craig, R. J. *The Millon clinical multiaxial inventory: A clinical research information synthesis*. (Routledge, 2013).
